# Dual or Mono Antiplatelet Therapy for the Prevention of Ischemic Stroke: A Literature Review

**DOI:** 10.7759/cureus.2847

**Published:** 2018-06-20

**Authors:** Ibrar Anjum, Tooba Kashif, Munis M Ahmed, Wafa Sohail, Mohsin Sarwar, Imran Khokhar

**Affiliations:** 1 Internal Medicine, The University of Texas MD Anderson Cancer Center, Houston, USA; 2 Cardiology, Sindh Medical College, Dow University of Health Sciences, Karachi, PAK; 3 Internal Medicine, King Edward Medical University, Lahore, PAK; 4 Dow Medical College, Dow University of Health Sciences, Karachi, PAK; 5 Student, Shifa College Of Medicine, Gujranwala, PAK; 6 General Surgery, Bronx Lebanon Hospital, New York, USA

**Keywords:** dual platelet therapy, ischemic stroke, aspirin and clopidogrel in stroke prevention, tia

## Abstract

Ischemic stroke is defined as a sudden loss of blood to the brain which results in deprivation of oxygen and other nutrients. It can be either a transient episode called as “transient ischemic attack” (TIA), or it could last longer than 24 hours giving rise to “infarction of tissues” in the central nervous system. Anti-platelet agents are widely used for the secondary prophylaxis of ischemic stroke, and amongst them, aspirin remains the drug of choice. In this literature review, we summarized the existing data regarding the ischemic type of strokes with particular attention to the use of antiplatelet agents for this purpose. The following review highlights the significance of the use of dual antiplatelet (aspirin and clopidogrel) regimen for the stroke prevention. The role of dual antiplatelet (aspirin and clopidogrel) in patients with a recent TIA (within 30 days) or severe stenosis (70%–99%) of a major intracranial artery, for 90 days, might be a beneficial approach.

## Introduction and background

Stroke is the fifth leading cause of death and is the major cause of disability in adults. Every year in the United States 795,000 suffer from the stroke [1]. The risk of having recurrent strokes and other adverse events is higher within 90 days of having transient ischemic attack (TIA) or stroke [2]. Usually, strokes are classified into two major subtypes:

1) An ischemic stroke which occurs due to obstruction of blood vessels by thrombi or embolus.

2) A hemorrhagic stroke which occurs due to rupture of blood vessels which could be due to intracerebral haemorrhage or subarachnoid haemorrhage.

Aspirin is commonly used drug for a variety of purposes, and it is often used as a prophylactic drug for any patient above 50 years of age. This gives the drug an almost equal misuse potential than any other over-the-counter (OTC) drugs. Aspirin potent antiplatelet drug works by inhibiting the production of cyclooxygenase and reducing thromboxane A2 levels, thus inhibiting thrombus formation within blood vessels [3]. It is essential to define the role of this drug for various atherosclerotic diseases. The purpose is to assess the use of antiplatelet regimens with special consideration as prophylaxis of stroke. Anti-platelet agents are widely used, and amongst them, aspirin remains the drug of choice. Even though it is broadly used, still the effectiveness remains questionable as studied by a metanalysis of the Antithrombotic Trialists [4]. This study has shown a 25% relative risk reduction for all kinds of vascular diseases and only 13% in cerebral ischemia [5]. Nevertheless, these drugs still have shown some extent of the required benefits. The use of aspirin was discouraged because of the side effects of gastric irritation, major and minor bleeding issues, and this led to the introduction of its competitor, ticlopidine. With time, even ticlopidine showed some side effects including diarrhoea and neutropenia, which led to the introduction of another popular drug used commonly nowadays in combination with aspirin as a dual antiplatelet regimen, clopidogrel [6]. Clopidogrel is a thienopyridine which further inhibits platelets aggregation by adenosine‐diphosphate (ADP) dependent mechanism [7].

## Review

Stroke is defined as inadequate blood flow to the brain that results in cell death. It can present with similar neurological dysfunction which could be either transient episode called “transient ischemic attack”, or it could last longer than 24 hours giving rise to “infarction of tissues” in the central nervous system. As per studies and surveys, about 80% of strokes are due to ischemic strokes, and the remaining 20% are hemorrhaging strokes [8].

Acute management of stroke depends on initial evaluation and stabilization of vital signs, e.g., airway, breathing, circulation, focused history and physical examination, non-contrast enhanced computed tomography (CT) scan or magnetic resonance imaging (MRI) of brain and timely restoration of blood flow to ischemic regions of the brain which has a narrow window period up to 4.5 hours for the administration of fibrinolytic tPA and up to 6 hours for mechanical thrombectomy depending on patient’s presentation and clinical expertise available [9]. Early and short time use of dual antiplatelet after TIA or stroke has supporting evidence for its benefits. Aspirin, when initiated within 48 hours of onset of stroke, has proven to be beneficial.

The Clopidogrel versus Aspirin in Patients at Risk of Ischemic Events (CAPRIE) was a famous study including almost 20,000 patients in which effectiveness of the response was analyzed amongst patients given clopidogrel (75 mg) versus aspirin (325 mg). The results showed a relative risk reduction of 8.7% but absolute risk reduction of 0.5% [10].

A trial on ischemic stroke was conducted by recruited 19,435 people within 48 hours of stroke onset. Half were randomized to receive either subcutaneous heparin or placebo and half were to receive aspirin or placebo. The study aimed to determine safety and efficacy of antithrombotic therapy for 14 days soon after stroke onset. In aspirin-treated patients who had fewer significant recurrent strokes within 14 days (2.8 vs 3.9) with no significant increased risk of haemorrhage (0.8 vs 0.9), the reduction of death and non-fatal stroke due to aspirin was significant (11.3 vs 12.4) [11].

Another more extensive study was conducted in a Chinese population. Chinese Acute Stroke Trial (CAST), a large randomized placebo-controlled trial to determine the efficacy of aspirin treatment when initiated within 48 hours of stroke onset and continued up to four weeks in the hospital, has also proven the efficacy of aspirin. There were significantly fewer recurrent ischemic strokes in an aspirin-allocated group than in the placebo-allocated group (1.6 vs 2.1) but slightly increased the risk of hemorrhagic stroke (1.1 vs 0.9). The absolute difference was 6.8 fewer cases per thousand in-hospital stroke or death in aspirin-treated patients. Both International Stroke Trial (IST) and CAST have proven the efficacy of aspirin in prevention of stroke when started within 48 hours of onset without a severe risk of hemorrhagic complications [12].

There is compelling evidence to support the use of dual antiplatelet therapy (aspirin and clopidogrel) in patients with acute coronary syndrome (ACS) with or without percutaneous coronary intervention (PCI). Indication for PCI is one year but there is no evidence to support the use of dual antiplatelet therapy for the primary prevention of ACS, or any cardioembolic stroke in atrial fibrillation. The potential benefits of dual antiplatelet risks versus benefits must be outweighed before using dual antiplatelet therapy for primary prevention of ischemic events [13].

The classification of ischemic stroke is based upon the mechanism of brain injury and the type of vessel involved. A Trial of Org 10172 in Acute Stroke Treatment (TOAST) classifies five subtypes of ischemic stroke: a) Large artery atherosclerosis which can be extra-cranial or intracranial, b) embolism from cardiac source, c) small vessel disease, d) stroke of other determined aetiology, and e) stroke of undetermined aetiology [14].

Multiple factors contribute to the pathogenesis of atherosclerosis including endothelial dysfunction, immunological, inflammatory cytokines, plaque rupture and its complications. Other independent risk factors for intracranial atherosclerosis are age, hypertension and diabetes [15]. Large Artery Intracranial Occlusive Disease (LAICOD), also known as Intracranial Atherosclerosis Disease (ICAD), is one of the most common causes of ischemic stroke worldwide and is associated with increased risk of recurrent stroke [16]. Intracranial atherosclerosis more commonlyaffects Asians, Hispanics and African Ancestry as compared to Caucasian. ICAD can often lead to TIA or ischemic stroke. ICAD major mechanism for ischemia is luminal narrowing and subsequent endothelial abnormalities contributing to plaque rupture and subsequent micro-embolisation leading to hypoperfusion and decreased washout of these emboli leading to ischemia of regions of the brain supplied by these arteries [17]. Extra-cranial and intracranial large vessel atherosclerosis itself accounts for 20% of ischemic strokes [18].

CHARISMA trial was conducted on 15,603 patients with multiple vascular risk factors or significant cardiovascular disease to receive treatment with either clopidogrel plus aspirin (low-dose) or placebo plus aspirin. Primary efficacy endpoint in this study was composed of myocardial infarction, stroke or death from any cardiovascular causes. The rate of primary efficacy endpoint was 6.8% in a group of clopidogrel and aspirin and 7.3% with placebo and aspirin (Relative risk, 0.93; 95% confidence interval (CI): 0.83-1.05; p = 0.22). The rate of the secondary endpoint which includes hospitalization for ischemic events was 16.7% in clopidogrel and aspirin, and 17.9% in placebo and aspirin (Relative risk, 0.92; 95% CI: 0.86-0.995; p = 0.04), however the rate of the primary composite, i.e. MI, stroke or death from any cardiovascular cause in patients with multiple vascular risk factors was 6.6% with clopidogrel and aspirin and 5.5% with placebo and aspirin. In a subgroup with significant atherothrombosis, the rate was 6.9% with dual antiplatelet and 7.9% with placebo and aspirin. Overall according to CHARISMA trial there was a beneficial outcome with dual antiplatelet therapy in a patient with significant atherothrombosis [19].

Several other clinical trials also determine the efficacy of dual antiplatelet therapy (aspirin and clopidogrel) which includes MATCH trial, a double-blinded placebo-controlled trial. A total of 7599 patients with recent stroke or TIA history were included to receive either aspirin or placebo in addition to clopidogrel. Duration of treatment and follow-up in this trial was 18 months. The primary endpoint was composed of ischemic stroke, myocardial infarction, worsening of peripheral arterial disease (PAD), vascular death or rehospitalization for acute ischemia (rehospitalization for the transient ischemic attack, angina pectoris, worsening of peripheral artery disease). The result findings of MATCH study were that 15.7% patients reached a primary endpoint in clopidogrel and aspirin group compared with 16.7% in clopidogrel alone group. Life-threatening bleedings were higher in aspirin and clopidogrel group versus clopidogrel alone group 2.6% versus 1.3% (95% CI: 0.6-1.9). Hence, MATCH study concludes that dual antiplatelet therapy in high-risk patients can lead to a non-significant decrease in major vascular events and associated with an increased risk of major bleeding [20]. However, the safety and efficacy of dual antiplatelet therapy need to be proven with further clinical trials. Several other risk factors must be taken into account while prescribing dual antiplatelet for stroke prevention depending on patient’s high-risk factors, for example, diabetes and multiple vascular disease and type of stroke whether it be a small vessel disease like lacunar stroke or large artery atherosclerosis. Dr Amarenco and Dr Donnan provided an insight into MATCH trial as the study population included was skewed with high-risk factors and 68% of which had diabetes mellitus. They also raised concerns on a large percentage of the population included in MATCH study, i.e. lacunar stroke which could impact the power and generalizability of study results to similar cerebrovascular disease. They further suggested that clinicians should exercise precaution while prescribing aspirin and clopidogrel in diabetic cerebrovascular disease patients [21].

Another double-blinded SPS3 trial was conducted on 3020 patients who had a recent lacunar stroke confirmed by MRI. Patients were randomly assigned to clopidogrel and placebo in addition to aspirin. They were followed up after a mean of 3.4 years. The data has shown the rate of recurrent stroke was not significantly reduced with both aspirin and clopidogrel as compared with aspirin alone (2.5% vs 2.7% per year), (hazard ratio (HR), 0.92; 95% CI: 0.72-1.16). The risk of recurrent ischemic stroke was reduced with dual antiplatelet therapy (HR: 0.82; 95% CI: 0.63-1.09). The risk of significant haemorrhage was doubled with dual antiplatelet therapy, 2.1% versus 1.1% per year (HR: 1.97; 95% CI: 1.41-2.71). The primary conclusion drawn from this study was that patients with lacunar stroke using dual antiplatelet therapy do not prevent the risk of recurrent stroke rather they increase the risk of significant haemorrhage and mortality [22].

Another clinical trial, Fast Assessment of Stroke and Transient Ischaemic Attack to Prevent Early Recurrence (FASTER), was conducted in 304 patients for the assessment of stroke and TIA to prevent early recurrence within 24 hours of those who had a recent TIA or minor stroke. The patients were assigned to receive clopidogrel or placebo, simvastatin or placebo in addition to aspirin and followed for 90 days. The trial was stopped earlier because of the failure of recruitment of patients at the prespecified rate. Data analysis showed that 7.1% of patients on clopidogrel and aspirin had a stroke compared with 10.8% of patients on aspirin and placebo within 90 days. Absolute risk reduction was -3.8% (95% CI: -9.4 to 1.9; p = 0.19). So, the conclusion is that the patients immediately after TIA or stroke have a high recurrence of stroke which can be reduced by using dual antiplatelet therapy. Its potential benefits cannot offset the risks of having a haemorrhage [23].

Another clinical trial CHANCE was conducted in 114 centres across the country in China. A total of 5170 patients were recruited into this trial with a criteria of within 24 hours of recent ischemic stroke or high-risk TIA to either receive clopidogrel and aspirin combination therapy (clopidogrel to a loading dose of 300 mg then followed by 75 mg for 90 days plus aspirin 75 mg for 21 days) or to placebo plus aspirin (75 mg for 90 days). The primary outcome of the trial was the stroke (ischemic or hemorrhagic) for 90 days follow-up in an intention-to-treat analysis. The secondary outcome was 8.2% of patients were in clopidogrel-aspirin group as compared with 11.7% of those in aspirin (HR: 0.68; 95% CI: 0.57-0.81; P&lt: 0.001). The rate of moderate to severe haemorrhage was 0.3% in both groups, and the rate of hemorrhagic stroke was also 0.3%. Hence, the result concluded that for the patients with minor ischemic stroke and TIA, when treated within 24 hours of the combination of clopidogrel-aspirin therapy, combination therapy (clopidogrel-aspirin) is superior to aspirin alone for preventing the risk of stroke and does not increase the risks of haemorrhage [24].

CARESS (Clopidogrel and Aspirin for Reduction of Emboli in Symptomatic Carotid Stenosis) is another double-blinded study with subjects having symptomatic or 50% or more stenosis. Microemboli signal (MES) detected by transcranial Doppler (TCD) was a feasible method used in this study to predict the risk of having stroke or TIA. Patients were screened with TCD, and on MES detection they were randomized to aspirin-clopidogrel combination group therapy or aspirin monotherapy group. They were screened again with TCD on day 2 and day 7. In a randomized group, 43.8% of dual-therapy was MES positive compared with 72.7% of monotherapy patients (relative risk reduction 39.8%; 95% CI: 13.8-58.0; p = 0.0046). MES frequency per hour was also reduced (compared with baseline) in dual therapy group by 61.6% on day 2 and 61.4% on day 7. There were four recurrent strokes and seven TIA in monotherapy group versus no stroke and four TIA in dual therapy. Hence, study results showed that in patients with symptomatic carotid stenosis dual antiplatelet therapy with aspirin and clopidogrel is more effective in reducing microembolization and the risk of recurrent stroke and TIA [25].

Multicentre trial, Stenting Aggressive Medical Management with or without Stenting in High-Risk Patients with Intracranial Stenosis (SAMMPRIS), was conducted in patients who had a recent TIA or stroke with 70%-99% stenosis of a major intracranial artery. The Patients were randomly assigned to intensive medical therapy (dual antiplatelet therapy, intensive management of vascular risk factors like diabetes, hypertension (HTN) and low-density lipoprotein (LDL) and lifestyle modification program) or PTAS (percutaneous transluminal angioplasty and stenting) using Wingspan stent. 5.8% of patients were enrolled in the medical treatment group, and 14.7% of patients in PTAS had a stroke or died within 30 days. During a mean follow-up of 32.4 months, 15% of patients in the medical treatment group and 23% of patients in the stenting had a primary endpoint composed of stroke or death. The occurrence of adverse events was also higher in PTAS than in medical group; stroke 26% versus 19%, major haemorrhage 13% versus 4% of patients. Findings of SAMMPRIS trial favor the use of aggressive medical management (aspirin 325 mg/d and clopidogrel 75 mg/d for 90 days) over percutaneous transluminal angioplasty and stenting using Wingspan in high-risk intracranial stenosis [26].

AHA/ASA guideline for the patients with a stroke or TIA caused by 50% to 99% stenosis of a major intracranial artery [27]:

a) Aspirin 325 mg/d is recommended in preference to warfarin (Class I; Level of Evidence B).

b) For patients with recent stroke or TIA (within 30 days) attributable to severe stenosis (70%–99%) of a major intracranial artery, the addition of clopidogrel 75 mg/d to aspirin for 90 days might be reasonable (Class IIb; Level of Evidence B).

c) For patients with a stroke or TIA attributable to 50% to 99% stenosis of a major intracranial artery, the data are insufficient to make a recommendation regarding the usefulness of clopidogrel alone, the combination of aspirin and dipyridamole, or cilostazol alone (Class IIb; Level of Evidence C).

d) For patients with a stroke or TIA attributable to 50% to 99% stenosis of a major intracranial artery, maintenance of Systolic Blood Pressure (SBP) below 140 mmHg and high-intensity statin therapy are recommended (Class I; Level of Evidence B).

e) For patients with a stroke or TIA attributable to moderate stenosis (50%–69%) of a major intracranial artery, angioplasty or stenting is not recommended given the low rate of stroke with medical management and the inherent periprocedural risk of endovascular treatment (Class III; Level of Evidence B).

f) For patients with stroke or TIA attributable to severe stenosis (70%–99%) of a major intracranial artery, stenting with the Wingspan stent system is not recommended as an initial treatment, even for patients who were taking an antithrombotic agent at the time of the stroke or TIA (Class III; Level of Evidence B).

g) For patients with stroke or TIA attributable to severe stenosis (70%–99%) of a major intracranial artery, the usefulness of angioplasty alone or placement of stents other than the Wingspan stent is unknown and is considered investigational (Class IIb; Level of Evidence C).

h) For patients with severe stenosis (70%–99%) of a major intracranial artery and recurrent TIA or stroke after the institution of aspirin and clopidogrel therapy, achievement of SBP below 140 mmHg is required.

## Conclusions

In this literature review, we summarized the existing data regarding antiplatelet agents used as a prophylaxis of ischemic type of strokes. We also briefly discussed the various types of stroke and antiplatelet agents as well as the role of inflammation in the mediation of atherosclerotic cascade. After the review of various studies, we have concluded that the potential benefits of dual antiplatelet regimen versus risks must be outweighed before using dual anti-platelets therapy for the primary prevention of ischemic events. The role of dual antiplatelet therapy (aspirin and clopidogrel) in patients with symptomatic large intracranial artery atherosclerosis who had a recent TIA or stroke, as per AHA/ASA guideline states that the addition of clopidogrel 75 mg/d to aspirin for 90 days might be a rational approach (Class IIb; Level of Evidence B) for the patients with recent stroke or TIA (within 30 days) attributable to severe stenosis (70%–99%) of a major intracranial artery.
